# Emotional and autonomic response to visual erotic stimulation in patients with functional hypothalamic amenorrhea

**DOI:** 10.3389/fendo.2022.982845

**Published:** 2022-12-02

**Authors:** Natale Maiorana, Agostino Brugnera, Valentina Galiano, Rosanna Ferrara, Barbara Poletti, Anna Maria Marconi, Emanuele Garzia, Nicola Ticozzi, Vincenzo Silani, Alberto Priori, Roberta Ferrucci

**Affiliations:** ^1^ Aldo Ravelli Research Center, Department of Health Science, University of Milan, Milan, Italy; ^2^ Department of Human and Social Sciences, University of Bergamo, Bergamo, Italy; ^3^ ASST-Santi Paolo e Carlo, Milan, Italy; ^4^ Department of Neurology and Laboratory of Neuroscience, Istituto Auxologico Italiano IRCCS, Milan, Italy; ^5^ Department of Health Science, University of Milan, Milan, Italy; ^6^ Department of Pathophysiology and Transplantation, Dino Ferrari Center, University of Milan, Milan, Italy

**Keywords:** functional hypothalamic amenorrhea (FHA), HRV variability analysis, RR variability, TAS 20, sex index, psychophysiology (all MeSH terms)

## Abstract

**Introduction:**

Functional hypothalamic amenorrhea (FHA) is a clinical condition associated with high levels of physiological and psychological stress ranging from weight loss to maladaptive behavior and coping skills. A reliable measure of the psychophysiological response to stress and the ability to cope with stimuli is heart rate variability (HRV). Through the sympathetic (SNS) and parasympathetic nervous system (PNS), the autonomic nervous system (ANS) promotes various changes in HRV that reflect the individual’s psychophysiological response to stress. FHA patients are characterized by high levels of PNS activation during psychological load, suggesting that parasympathetic hyperactivation could be a pathology marker.

**Methods:**

In the present study, we examine changes in HRV during observation of erotic, neutral, and disgusting images in 10 patients with FHA [(mean ± S.D.) age: 26.8 ± 5.9] and in 9 controls (age: 25.4 ± 6.4; BMI: 22.47 ± 2.97) to assess the differential activation of PNS and SNS between FHA patients and controls matched for age and without other clinical conditions.

**Results:**

Our results showed that FHA patients had significantly higher HRV activation while observing high emotional value images and not during the observation of neutral images confirming a parasympathetic hyperactivation.

**Discussion:**

HRV and cognitive and psychological testing, could provide new insights into understanding such a clinically understudied condition and provide further tools for clinical diagnosis and treatment.

## Introduction

Functional hypothalamic amenorrhea (FHA) is a condition in which an abnormality in gonadotropin-releasing hormone (GnRH) secretion leads to impairments in follicle-stimulating hormone and luteinizing hormone (1, 2). FHA is diagnosed after other causes have been ruled out, and its cause appears to be pulsatile hypothalamic gonadotropin-releasing hormone (GnRH) dysfunction with consequences for follicle stimulating hormone (FSH), luteinizing hormone (LH), and estradiol levels. FHA accounts for 30% of cases of secondary amenorrhea (3). Psychologically, FHA is associated with high levels of stress, excessive physical activity, maladaptive eating disorders, and weight loss (4). In recent years, many studies have focused their attention on the relationship between FHA and psychological variables that influence coping strategies in FHA patients (5). There is ample evidence that stress affects endocrine networks, in particular overactivity of the hypothalamic-pituitary-adrenal (HPA) axis has been observed in women with FHA, leading to elevated cortisol levels (6). The relationship between FHA and psychological factors is circular: psychological factors can lead to FHA and at the same time FHA has a significant impact on women’s psychological well-being (7). A reliable measure of the psychophysiological response to stress and the ability to cope with stimuli is heart rate variability (HRV) (8). Changes in heart rate are an indicator of the adaptation of the cardiovascular and nervous system to the environmental requests (9). HRV is defined as the variation of the heartbeats in the time interval and is measured considering the variation in the beat to beat interval (10). A reliable index of HRV is the standard deviation between beat intervals (SDNN) calculated by excluding technical and physiological artifacts. SDNN is calculated by the square root of the total variance in the ECG recording. Low SDNN was found in patients diagnosed with PTSD, low SDNN reflect lower activity of the PNS and a reduced physiological response to cope with stress (11–13).

Another index used to characterize HRV is the root mean square of the successive difference (RMSSD), calculated as the proportion of NN intervals larger than a given threshold (14). RMSSD can be used to estimate the vagal contribution in HRV (15). RMSSD was associated with higher levels of anxiety and depression (16).The two main branches of the autonomic nervous system (ANS), the sympathetic nervous system (SNS) and the parasympathetic nervous system (PNS) influence HRV. Through the SNS and PNS, the ANS promotes various changes in HRV that reflect the individual’s psychophysiological response to stress. In particular, retraction of the PNS causes activation of the SNS, resulting in the so-called fight or flight response, which is an automatic physiological response to stressful events (17). During the stress response, the hypothalamic-pituitary axis (HPA) triggers endocrine changes such as the release of corticotropin hormone from the hypothalamus (18). Several findings suggest that an adequate level of PNS activation is a protective factor in the development of mental illness (19). It was observed that individuals with high resting state HRV were characterized by less perceived stress and a high capacity for emotional self-regulation than individuals with lower resting state HRV (13, 20). High resting state HRV is associated with a predominant role of PNS over SNS. By vagal nerve activation the PNS decrease heart rate (21). The action of PNS is fast and given the short times range used in HRV measures HRV represents the activation or withdrawal of PNS (21–23). To assess the response of the ANS to stress, it is possible to assess HRV reactivity by comparing baseline HRV to HRV indices when faced with an emotionally charged stimulus. In this sense, high HRV reactivity may reflect maladaptive activation of the PNS, making subjects less responsive to the stressor (24), resulting in a delayed response. It was found that FHA patients showed increased HRV reactivity in response to stress stimulation than controls, FHA patients were characterized by increased parasympathetic activation without the typical concomitant change in heart rate due to sympathetic activation in response to orthostatic stress (25).

Galetta and colleagues (2003) (26) found a change in HRV in a sample of patients with anorexia nervosa characterized by hyperactivation of the parasympathetic nervous system and suggested that HRV and diastolic analysis are useful metrics to assess the severity of the pathology. Similar results were found by Bomba and colleagues in 2014 (5), where an HRV comparison between FHA and AN patients showed similar patterns with increased parasympathetic nervous system activation during a 24-hour recording. When subjected to cognitive load during the Stroop color word test, FHA patients exhibit low heart rate, low systolic and diastolic blood pressure, indicating hyper vagal tone that does not permit task completion, resulting in poorer performance than controls (27). However, it remains unclear whether HRV and HRV-reactivity can be a psychophysiological marker for FHA. It should be noted that FHA patients are characterized by an atypical vagal response and consequent SNS activation than the general population (25, 28), which may indicate neurophysiological and psychophysiological correlates of the disease useful in daily clinical practice.

FHA patients can be also affected by sexual dysfunction caused by hypoestrogenism causing impairment in genital receptivity and reduction in libido (29–31). However due to the complex interaction between mood disorders and hormonal dysfunction to date it is still unclear whether sexual dysfunction in FHA is due to hormonal imbalance or to maladaptive psychological behavior and coping strategies (31). To date the relationship between sexual dysfunction, FHA, neuroendocrine and psychological functioning remain unclear and no study investigated the role of physiological activation related to sexual arousal in patients with FHA.

In the present study, we examine changes in HRV during observation of erotic, neutral, and disgusting images in patients with FHA to assess the differential activation of PNS and SNS between FHA patients and controls.

## Methods

### Sample

A total of 19 women took part in the experiment. Ten participants were patients with FHA [(mean ± S.D.) age: 26.8 ± 5.9; BMI: 21.23 ± 2.55] and the other 9 participants were healthy subjects (age: 25.4 ± 6.4; BMI: 22.47 ± 2.97) with no history of amenorrhea or other significant clinical conditions. All participants declared to be heterosexual. All participants had no history of neuropsychiatric disorders and had normal or near-normal visual acuity. All participants were non-smokers or light smokers with a daily consumption of less than 25 cigarettes (**Table 1**). No subject reported drug or alcohol abuse. The study was approved by the Institutional Ethics Committee (3415/2018) and was conducted in accordance with the Declaration of Helsinki. All participants provided written informed consent to participate in this study.

**Table 1:** Demographic, anthropometric and consumption habits of the experimental sample.

Participant	Age	Weight (Kg.)	Height (cm.)	Physical activity (min/day)	Smoker	Cigarettes consumption(Cigarettes/day)	Coffee consumption(N. coffee/day)	Alcohol consumption(units/week)
FHA patient 1	31	50	160	90	no	0	2	1.5
FHA patient 2	24	51	163	240	no	0	2	0
FHA patient 3	30	62	157	90	no	0	5	0
FHA patient 4	24	58	160	240	no	0	1	4.5
FHA patient 5	30	60	152	180	no	0	3	0
FHA patient 6	40	51	164	150	no	0	1	1.5
FHA patient 7	23	57	168	0	no	0	0	3
FHA patient 8	21	55	170	300	No	0	1	1.5
FHA patient 9	23	58	166	300	No	0	1	1.5
FHA patient 10	22	51	158	300	no	0	1	0
CONTROL 1	26	75	165	0	no	0	0	0
CONTROL 2	22	66	170	300	si	4	2	3
CONTROL 3	20	52	163	150	no	0	2	4.5
CONTROL 4	21	55	163	350	no	0	0	3
CONTROL 5	20	57	160	180	no	0	4	3
CONTROL 6	25	68	180	240	si	2	3	4.5
CONTROL 7	40	70	160	90	no	0	3	3
CONTROL 8	30	50	155	300	si	4	2	4.5
CONTROL 9	25	55	165	90	no	0	1	3

### Protocol

Each experimental session started with 5 minutes cardiac baseline recording where the subject was in seated resting state position.

**Figure 1:**
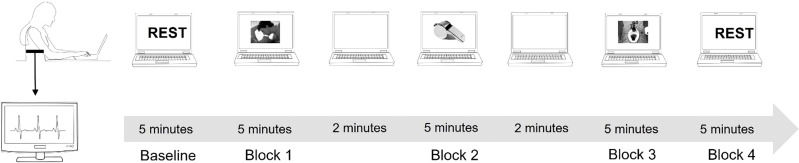
Experimental procedure.

Participants were asked to closely watch a slideshow that featured erotic images, neutral images, or disgusting images. The slide show was divided into three blocks, each block consisted of images of the same category (e.g. erotic, neutral, disgusting), and the order of the blocks was randomly chosen between the participants. A two-minute blank screen separated the image blocks. During this time, participants were asked to rate the images they had just seen. Rating was performed according to the IAPS database rating system by compiling VAS scales of how satisfied vs. dissatisfied, calm vs. excited, or controlled vs. in control based on the images just viewed. For each category, 24 images were selected from the IAPS database (32). Each image stayed on screen for about 5 seconds and was repeated twice in one block. The order of the blocks was randomized between participants. (**Figure 1**). After the cardiac recording was completed, participants completed a neuropsychological assessments composed by MoCA Test: a screening assessment for detecting cognitive impairment (24); Stroop Color Word Test (33) to assess the ability to inhibit cognitive interference; a computer-based Go-No-Go test to assess sustained attention and response control; Simple Reaction Times to assess the functioning of global attention were performed. After completing the neuropsychological assessment phase, the TAS-20 (34) and Hendrick Sexual Attitude Scale (35) were administered to assess the presence of marked alexithymia or sexual attitudes that might explain cardiac response to the specific stimuli presented.

### ECG data

Electrocardiogram (ECG) data were collected with Pulse, developed by STMicroelectronics and manufactured by MR&D (Italy). Pulse is a wearable device with a sampling rate of 256 Hz. The device was attached to the center of the participant’s chest with an elastic band containing electrodes. The center of the chest corresponds to lead 1 of the standard 12-lead ECG. The ECG was recorded in one session divided into 5 blocks, with the first block consisting of the baseline recording of the subjects’ resting state for 5 minutes. The second, third, and fourth blocks were registered while the subject watched the slide show, each block of the slide show lasting about 5 minutes. Each slideshow block was separated by a two-minute blank slide. The last block was the recovery phase recording and lasted 5 minutes. The pulse sensor filtered the signal with a bandpass filter (0.05 to 40 Hz). The raw ECG signal saved in European Data File format (EDF+) was then transferred to Kubios HRV software 3.1 (36) for HRV analysis. A QRS detector algorithm was used to extract the beat-to-beat RR intervals from the ECG data. The ECG was also examined visually to detect and correct for artifacts such as missing or extra beats. All ECG recordings with an artifact rate of less than 5% were included in the data analysis. Very low frequency components (<0.04 Hz) were removed in a pre-processing process using a detrending approach based on smoothness priors (36, 37).

**Figure 2:**
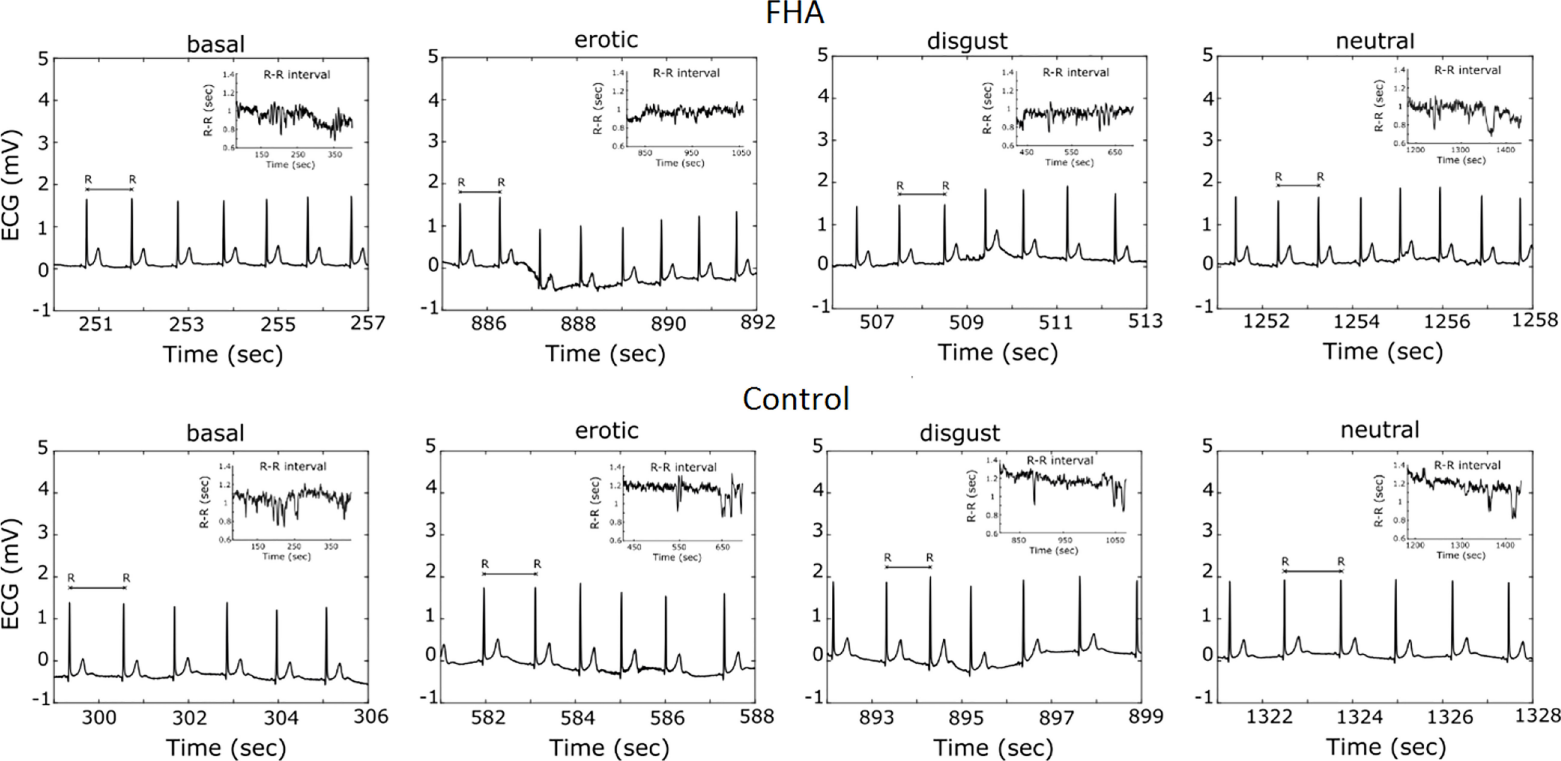
Example of Electrocardiogram recording and R-R- interval extraction in a casually selected FHA patient and Control subject.

We focused on mean RR interval: the mean interval, measured in ms, between RR peaks (**Figure 2**), LnHFP: the logarithm of the fast Fourier transform of high frequency power; SDDN: Mean of the standard deviations of all NN intervals for each segment of the recording; RMSSD. The root mean square of successive differences between normal heartbeats (RMSSD).

### Statistical analysis

All analyzes were performed with SPSS 26.0 (IBM, 2019). The normality of the data was assessed using the Shapiro-Wilk normality test, the data were normally distributed, therefore the data were analyzed using a parametric test. Within-subject differences in HRV variables recorded while subjects viewed different image blocks were compared to baseline using paired-sample t-tests. To better examine the differences between the groups, indices of difference in HRV variables were also calculated by subtracting the HRV values ​​recorded at baseline from the HRV values ​​recorded during the experimental blocks, and then compared the differences in cardiac indices between groups using one-way ANOVAs.

One-way ANOVAs were also used to assess differences in psychological and neuropsychological data between groups. For all analyses, a p-value of 0.05 was considered significant.

## Results

### ECG data

**Figure 3:**
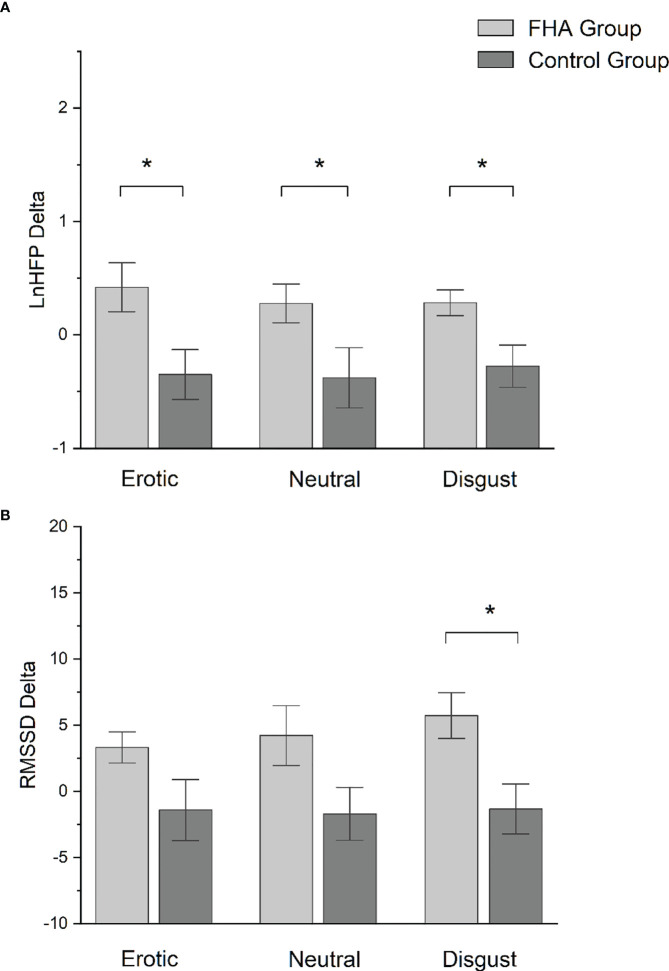
**(A)** Bar-chart representing lnHFP delta in FHA group and control group. Error bars represent standard errors. *p value < 0.05; **(B)** Bar-chart representing RMSSD delta in FHA group and control group. Error bars represent standard errors. *p value < 0.05.

**Figure 4:**
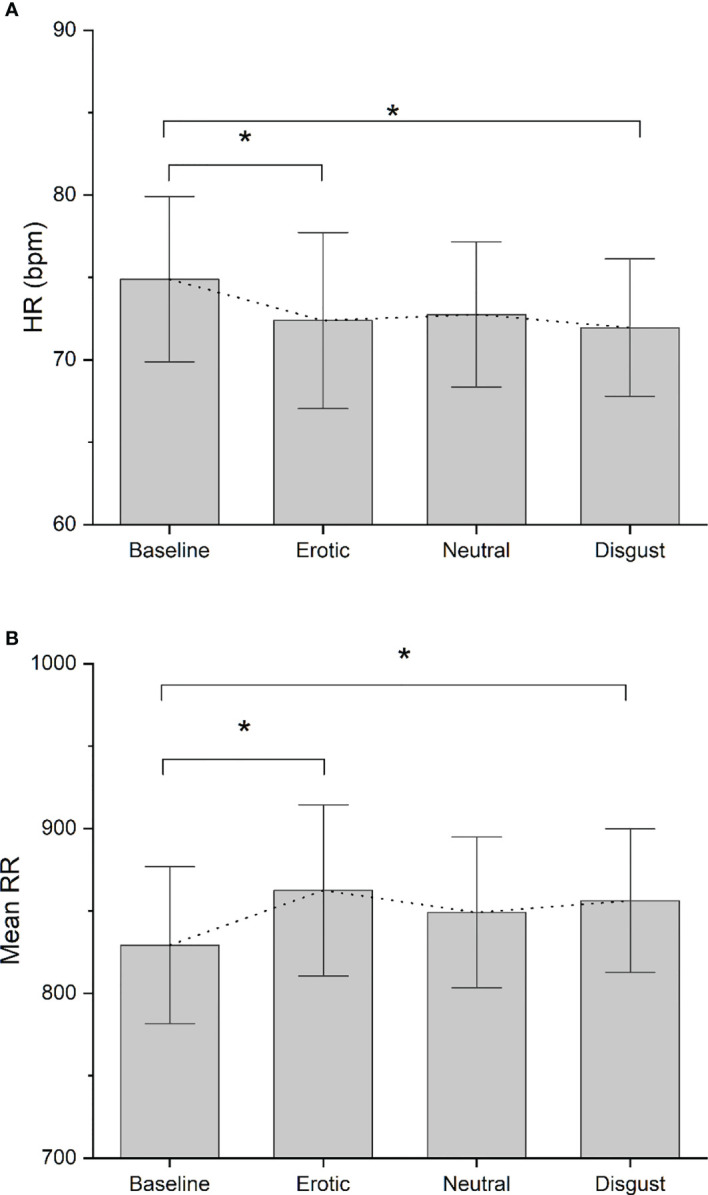
**(A)** Bar-chart representing Mean HR recorded in each experimental block in FHA group. Error bars represent standard errors. Dot-line connects the mean value recorded in each block. *p value < 0.05; **(B)** Bar-chart representing Mean RR recorded in each experimental block in FHA group. Error bars represent standard errors. Dot-line connects the mean value recorded in each block. *p value < 0.05.

No significant differences were found between FHA patients and controls in baseline ECG data (p>0.05). Comparing HRV difference indices between FHA and control group, we found significant differences in LnHFP during erotic block [(mean ± standard deviation) FHA vs Controls: 0.41 ± 0.68 vs -0.35 ± 0.66); F_1,17_ = 6.175 p =0.02], in neutral block [FHA vs Controls, 0.28 ± 0.54 vs -0.38 ± 0.79; F _1,17_ = 4.528, p = 0.04)] and in disgust block [FHA vs controls: 0.28 ± 0.35 vs -0.27 ± 0.59; F _1,17_ = 6.919, p = 0.01] (**Figure 3A**). In addition, ANOVA showed differences in RMSSD in disgust block between the FHA and control groups [FHA vs Controls: 5.73 ± 5.48 vs -1.31 ± 5.66; F _1,17_ = 7.622, p = 0.01] (**Figure 3B**). No significant differences between groups were found in other HRV variables indexes calculated (all p > 0.05). Paired sample t-tests showed that mean heart rate decreased in the FHA group during observation of erotic images [baseline vs erotic, 74.88 ± 15.86 vs. 72.38 ± 16.89; t = 3.115 p= 0.01] and disgusting images compared to baseline [baseline vs. disgust:74.88 ± 15.86 vs. 71.95 ± 13.17; t= 2.807 p=0.02] (**Figure 4A**). The mean RR increased in the FHA group during observation of erotic images [baseline vs. erotic: 829.24 ± 150.83 vs. 862.47 ± 164.25; t = -3.540 p = 0.01] and during observation of disgusting images [baseline vs. disgust: 829.24 ± 150.83 vs. 856.19 ± 137.65; t =-3.606 p = 0.01] compared to baseline (**Figure 4B**). RMSSD also increased in the FHA group during observation of erotic images [baseline vs. erotic: 38.80 ± 16.73 vs 42.13 ± 16.97; t = -2.829 p = 0.02] and during observation of disgusting images [baseline vs. disgust:38.80 ± 16.73 vs 44.53 ± 19.26; t = -3.305 p = 0.01] compared to baseline. LnHFP increased in the FHA group compared to baseline only during observation of disgusting images [baseline vs. disgust: 6.13 ± 0.86 vs 6.41 ± 0.73; t = -2.533 p = 0.03]. In addition, we found that SDNN decreased in the control group during observation of erotic images compared to baseline (baseline vs erotic 61.50 ± 21.38 vs 49.49 ± 17.09; t = 3.493 p = 0.01). No significant differences were found analyzing other cardiac data in control group (all p > 0.05).

Neutral images had no effect on the ECG data. No significant differences were found between patient and control group in the explicit rating of images for any of the image categories presented (all p > 0.05) (**Table 2**).

**Table 2:** Cardiological data in Functional Hypothalamic Amenorrhea group (FHA) and control group (control) for each picture block.

	Baseline		Erotic pictures		Neutral Pictures		Disgusting Pictures	
	FHA	CONTROL	FHA	CONTROL	FHA	CONTROL	FHA	CONTROL
Mean HR	74,88 ± 15,86	76,23 ± 8,25	72,38 ± 16,89	73,39 ± 10,31	72,75 ± 13,92	73,99 ± 10,51	71,95 ± 13,17	74,10 ± 10,50
Mean RR	829,24 ± 150,83	796,80 ± 102,13	862,47 ± 164,25	835,12 ± 141,78	849,06 ± 144,66	829,03 ± 145,04	856,19 ± 137,65	827,70 ± 144,30
RMSSD	38,80 ± 16,73	35,86 ± 12,96	42,13 ± 16,97	34,45 ± 15,72	43,03 ± 18,38	34,16 ± 12,55	44,53 ± 19,26	34,53 ± 14,13
LnHFP	6,13 ± 0,86	6,10 ± 0,79	6,55 ± 0,74	5,75 ± 0,91	6,40 ± 1,03	5,72 ± 0,88	6,41 ± 0,73	5,82 ± 0,49
SDNN	62,04 ± 16,68	61,50 ± 21,38	54,38 ± 15,07	49,49 ± 17,09	59,05 ± 18,70	59,86 ± 20,26	61,62 ± 14,13	52,95 ± 18,71

Mean heart rate (Mean HR); Mean Interval measured between RR peaks (Mean RR); Root mean square of successive differences between normal heartbeats (RMSSD); logarithm of the Fast Fourier Transform of the High Frequency Power (LnHFP); Mean of the standard deviations of all the NN intervals for each segment of the recording (SDNN). Data are expressed ad mean ± Standard Deviation.

### Neuropsychological and psychological assessment

We calculated error interference effects in the Stroop color word test using the formula {Error Interference Sheet - [(Error Reading Sheet + Error Naming Sheet)/2]} (25) and then compared the Error Interference Index between FHA and control group. Compared to controls FHA patients showed a higher error-interference index than controls in Stroop’s test [(mean ± S.D) Controls vs FHA: 0.00 ± 0.00 vs 0.30 ± 0.42); F_1,17_Rosalyn= 4.530 p = 0.048].

FHA patients made more errors than controls on the go-no-go task (Controls vs FHA: 0.22 ± 0.44 vs 1.30 ± 1.25; F_1,17_ = 5.975 p = 0.026] (**Table 3**).

**Table 3:** Results of Stroop Color Word Test, GO-NO-GO Test, Simple Reaction Times, Toronto Alexithymia Scale (TAS-20), Hendrick Sexual Attitude Scales in FHA (Functional Hypothalamic Amenorrhea group) and in controls (control group).

	STROOP CW Test		GO-NO-GO		SIMPLE REACTION TIMES	TAS-20	Hendrick Sexual Attitude Scales			
	Errors	Time	Errors	Time			Permissiveness	Practice	Communion	Instrumentality
FHA	0,5 ± 0,70	27,55 ± 8,85	1,3 ± 1,25	472,75 ± 122,22	321,48 ± 42,42	43,55 ± 14,70	3,60 ± 0,55	1,56 ± 0,48	2,16 ± 0,58	3,22 ± 0,60
CONTROL	0,11 ± 0,33	24,97 ± 2,80	0,22 ± 0,44	479,25 ± 52,01	314,38 ± 18,90	52,88 ± 9,49	3,23 ± 0,36	1,47 ± 0,46	1,85 ± 0,41	3,24 ± 0,77

Data are expressed as mean ± Standard Deviation.

No significant differences in simple reaction times were found between groups (all ps > 0.05).

No significant difference was found in the TAS-20 scores, Hendrick Sexual Attitude Scales, between the FHA and control groups (all ps > 0.05).

## Discussion

This study investigated autonomic responses to the observation of arousing pictures in a sample of patients with FHA and in controls. Our results show that FHA patients have significantly higher HRV activation during observing of high emotional value images (erotic and disgusting images) compared to baseline and not during observing of neutral images.

To the best of our knowledge this is the first study assessing the HRV response to the observation of emotional pictures in FHA patients.

High HRV is associated with activation of PNS (38), in our sample it seems that FHA patients have higher PNS activation than controls during observation of images characterized by emotional valence.

While baseline HRV did not differ between FHA patients and controls, FHA patients showed higher HRV reactivity (39). HRV reactivity reflects parasympathetic activation in response to external events (19). While resting HRV has been described as adaptive, reflecting functional autonomic regulation, high HRV reactivity may reflect maladaptive activation of the parasympathetic nervous system (38, 40). Indeed, increased parasympathetic nervous system activation might reflect decreased responsiveness with delayed response mobilization to meet environmental demands (41). During challenging stimulation, a subject with a decrease in HRV may be more responsive and tend to respond with marked activation of the sympathetic nervous system to face the external stimuli (41).

Changes in HRV indices during exposure of IAPS images were studied by Kwang-Ho Choi and colleagues (42) who found a positive correlation between HRV and valence and a negative correlation between HRV and dominance. However, Kwang-Ho Choi and colleagues found HRV variations only in response to images characterized by a negative valence with a strong activation value. In our sample, controls showed no parasympathetic activation during observation of both negative and positive-scored images, while FHA patients showed higher HRV reactivity to both positive and negative-scored images. HRV reactivity in FHA patients could represent hyperactivation of the parasympathetic nervous system that needs further investigation. In addition, studies have found PNS activation during sexual arousal or disgust responses (43, 44), suggesting a possibly lower threshold for PNS activations than controls due to PNS hypertonicity in FHA patients.

Differences between FHA and controls in HRV could be due to the effects of hormone levels on cardiac activity due to parasympathetic activation (45). Leptin perfusion in the arcuate nucleus of the hypothalamus in rats was found to increase sympathetic nervous system activity (46). It should be possible that in FHA, low levels of leptin affect the neurons of the arcuate nucleus, resulting in decreased sympathetic nervous system activity and increased parasympathetic activity.

In addition, GnRH levels might affect the preoptic hypothalamus and arcuate nucleus, which play a central role in PNS activity (46–48). The preoptic hippocampus in ewes has been found to be sensitive to GnRH and that GnRH levels are elevated during the follicular phase of the oestrus cycle. FHA is associated with low calorie intake and less available energy for the organism is (49), parasympathetic hypertension leading to heart rate slowdown and bradycardia could be a protective mechanism to adapt to starvation and reduce energy expenditure (50, 51). A disease associated with low energy expenditure that overlaps with FHA is anorexia nervosa, Galetta and colleagues (2003) (26) found an alteration in HRV in a sample of patients with anorexia nervosa characterized by hyperactivation of the parasympathetic nervous system during a 24h recording and suggested that HRV and diastolic analysis might provide a useful measure for assessing the severity of pathology.

Similar results to those of Galetta and colleagues (26) were found by Bomba and colleagues in 2014 (52) where HRV comparison between FHA and AN patients showed similar patterns during a 24h recording with increased activation of the parasympathetic nervous system. Taking these results together, it is possible that FHA patients exhibit altered parasympathetic activation that could reflect a possible continuum between these two pathologies, with hormonal and psychological dysfunctions of the patients (5) together with the energy available to the organism playing a modulating role. Like anorexia nervosa, FHA is a multifaceted disease characterized by a complex interaction between psychological and physiological factors. FHA patients show similar psychopathological patterns as anorexia nervosa patients, but at lower levels that do not meet the criteria for a clinical diagnosis (53). Differences between HRV in FHA and controls could be due to complex interactions between hormone levels, available energy and ability to cope with excitatory stimuli and everyday stressors (5). The results of the psychological questionnaire administered to our participants showed no significant differences in terms of depression, alexithymia and sexual attitudes. Our results are consistent with studies describing that FHA patients do not meet the criteria for a psychopathological diagnosis (4, 5). However, more research is needed to better examine psychological variables that may influence such findings, both in measuring personality traits and in recording clinical interviews and psychological history.

Regarding cognitive performance, we found that FHA patients made more errors and showed stronger interference effects on the Stroop CW test than controls. In the Stroop CW, subjects are asked to suppress the automatic reading of the presented words and to name the ink color in which the words are written (33). The Stroop CW test is a challenging test and is associated with increased systolic blood pressure and heart rate (54, 55) in response to cognitive load and mental stress (27). Similar to our results, Gallinelli and colleagues found that FHA patients had lower values ​​on the Stroop CW test along with lower blood pressure and slower heart rate during the test (27). The Stroop CW test is physiologically linked to the anterior cingulate cortex (56), which is heavily involved in attentional processes. Furthermore, the anterior cingulate cortex is connected to the hypothalamus and may play a role in activating the hypothalamic-pituitary-adrenal axis (55, 56) when one is under high cognitive load or a stressful state (55). The influence of the anterior cingulate cortex on the hypothalamic-pituitary-adrenal axis may explain the effects of cognitive load and stress on ANS activation (57). However, regarding our results the link between PNS activation and performance at the Stroop CW test remain at a speculative level only since we did not recorded cardiological data during the execution of the Stroop CW Test.

Similar to the results of the Stroop color word test, we found that FHA patients made more errors on the Go-No-Go task, a cognitive-behavioral task that assesses the ability to inhibit responsive behavior (58). In rats performing a Go-NO-Go task with food reward, it was observed that increased activity in orexin neurons in the medial hippocampus was associated with their greater accuracy (59). In addition, orexin has been associated with food cravings, sympathetic activation and effects on the hypothalamic-pituitary-gonadal axis in *in vitro* and *in vivo* studies (60, 61).In humans, orexin is implicated in appetite, behavior, and psychophysical activity *via* regulation of reproductive and stress hormone secretion (62). Orexin has been found to affect eating behavior and stress response in anorexia nervosa patients, affect the hypothalamo-pituitary-adrenal (HPA) and hypothalamo-pituitary-gonadal axes, and activate the sympathetic nervous system in anorexia nervosa patients (62). Despite these preliminary results, future studies should investigate the role of orexin in FHA to better explain the complex neurophysiological and neuropsychological phenomena associated with the disease. To date, little is known about the relationship between HRV and FHA, and no study has focused on HRV response to external stimulation with emotionally fluctuating images in FHA patients. Our study presents certain limitation, statistical power could have been affected by the limited sample size, furthermore we did not have information about hormonal and metabolic profile of the sample, these data could be useful to better characterize differences between FHA patients and control subjects.

Despite the mentioned limitations, the results of our study suggest that FHA patients may have a different cardiac response than healthy controls in response to emotionally activating stimulation. Further studies are needed to clarify the role of emotional external stimulation and cognitive load in ANS activation in FHA patients. Our results suggest that it is possible to use images from the IAPS as an excitatory, reliable stimulation method to elicit HRV changes and assess parasympathetic activation. FHA is a complex disease with neuroendocrine and psychophysiological correlates. HRV, along with cognitive and psychological testing, could provide new insights into understanding such a clinically understudied condition and provide further tools for clinical diagnosis and treatment. Considering the link between FHA, mental illness (63) and the neuroendocrine (43) and psychophysiological (4, 52) features of FHA, clinical interventions should be characterized by multidisciplinary approaches. Studies on psychotherapeutic interventions for stress reduction and development of adaptive coping skills have shown promising results with cognitive behavioral therapy and hypnosis (64, 65), it might be useful to implement approaches with biofeedback based on HRV.

## Data availability statement

The raw data supporting the conclusions of this article will be made available by the authors, without undue reservation.

## Ethics statement

The studies involving human participants were reviewed and approved by San Paolo Hospital review board. The patients/participants provided their written informed consent to participate in this study.

## Author contributions

NM, design of the work, collected the data, performed the analysis, and wrote the paper. AB, RosF, BP, VS, NT, and EG, contributed data or analysis tools, performed the analysis, and revised the paper. VG and AM, collected the data and revised the paper. AP and RobF, design of the work, contributed data analysis, wrote, and revised the paper. All authors contributed to the article and approved the submitted version.

## Acknowledgments

This study was partially supported from Aldo Ravelli Research Center for Neurotechnology and Brain Therapeutics.

## Conflict of interest

The authors declare that the research was conducted in the absence of any commercial or financial relationships that could be construed as a potential conflict of interest.

## Publisher’s note

All claims expressed in this article are solely those of the authors and do not necessarily represent those of their affiliated organizations, or those of the publisher, the editors and the reviewers. Any product that may be evaluated in this article, or claim that may be made by its manufacturer, is not guaranteed or endorsed by the publisher.
